# Supplementary material for: A Critical View of the Application of the APEX Software (Aqueous Photochemistry of Environmentally-Occurring Xenobiotics) to Predict Photoreaction Kinetics in Surface Freshwaters
Source: Molecules. 2019 Dec 18;25(1):9. doi: 10.3390/molecules25010009 (PMC7017383; doi:10.3390/molecules25010009)
Supplement: Supplementary file 1 [file molecules-25-00009-s001.zip › 3.2.4_gcc-4.4.0.zip!/3.2.4_gcc-4.4.0/3.2.4_gcc-4.4.0/doc/octave/html/interpreter/Preface.html]

Preface - Untitled


# Untitled

Next: Introduction,
Previous: Top,
Up: Top


---

## Preface

Octave was originally intended to be companion software for an
undergraduate-level textbook on chemical reactor design being written by
James B. Rawlings of the University of Wisconsin-Madison and John
G. Ekerdt of the University of Texas.

Clearly, Octave is now much more than just another `courseware' package
with limited utility beyond the classroom. Although our initial goals
were somewhat vague, we knew that we wanted to create something that
would enable students to solve realistic problems, and that they could
use for many things other than chemical reactor design problems.

There are those who would say that we should be teaching the students
Fortran instead, because that is the computer language of engineering,
but every time we have tried that, the students have spent far too much
time trying to figure out why their Fortran code crashes and not enough
time learning about chemical engineering. With Octave, most students
pick up the basics quickly, and are using it confidently in just a few
hours.

Although it was originally intended to be used to teach reactor design,
it has been used in several other undergraduate and graduate
courses in the Chemical Engineering Department at the University of
Texas, and the math department at the University of Texas has been using
it for teaching differential equations and linear algebra as well. If
you find it useful, please let us know. We are always interested to
find out how Octave is being used in other places.

Virtually everyone thinks that the name Octave has something to do with
music, but it is actually the name of a former professor of mine who
wrote a famous textbook on chemical reaction engineering, and who was
also well known for his ability to do quick `back of the envelope'
calculations. We hope that this software will make it possible for many
people to do more ambitious computations just as easily.

Everyone is encouraged to share this software with others under the
terms of the GNU General Public License (see Copying). You are
also encouraged to help make Octave more useful by writing and
contributing additional functions for it, and by reporting any problems
you may have.

- Acknowledgements- How You Can Contribute to Octave- Distribution
